# Supplementary material for: A novel function for the sperm adhesion protein IZUMO1 in cell–cell fusion
Source: J Cell Biol. 2022 Nov 17;222(2):e202207147. doi: 10.1083/jcb.202207147 (PMC9671554; doi:10.1083/jcb.202207147)
Supplement: Table S1 — shows GCS1/HAP2 and IZUMO1 induce syncytia formation. [file JCB_202207147_TableS1.docx]

| **Table S1. GCS1/HAP2 and IZUMO1 induce syncytia formation.** Multinucleation was determined in four independent experiments in BHKs expressing myristoylated GFP (myrGFP), GCS1/HAP2, IZUMO1 or JUNO. Cells with 2 or more nuclei are considered multinucleated. Related to Figure 1. | | | | | |
| --- | --- | --- | --- | --- | --- |
|  | **Number of cells with:** | | | | |
|  | **1 nucleus** | **2 nuclei** | **3 nuclei** | **4 nuclei** | **5 nuclei** |
| **myrGFP** | 3462 | 180 | 8 | 0 | 0 |
| **GCS1/HAP2** | 2202 | 439 | 39 | 1 | 0 |
| **IZUMO1** | 2740 | 399 | 50 | 15 | 1 |
| **JUNO** | 3400 | 232 | 3 | 0 | 0 |
