# Supplementary material for: A novel function for the sperm adhesion protein IZUMO1 in cell–cell fusion
Source: J Cell Biol. 2022 Nov 17;222(2):e202207147. doi: 10.1083/jcb.202207147 (PMC9671554; doi:10.1083/jcb.202207147)
Supplement: Table S2 — lists primers used for this study. [file JCB_202207147_TableS2.docx]

| **Table S2. Primers used for this study** | | |
| --- | --- | --- |
| **Primer name** | **Sequence 5'->3'** | **Description** |
| ZP2 F EcoRI | GCGAATTCATGGCGAGGTGGCAGAGGAAAG | Forward primer for cloning *mZP2* into pIZT and pCAGGS with EcoRI |
| ZP2 R NotI | TGGGCGGCCGCCGTGATTGAACCTTATAGTTCTTTTC | Reverse primer for cloning *mZP2* into pIZT with NotI |
| ZP2 R NheI | CTTACGCTAGCTCAATGGTGATGGTGATGATG | Reverse primer for cloning *mZP2* into pCAGGS with NheI |
| Izumo1 F EcoRI | CCGGGAATTCGGTCGACTGGATCC | Forward primer for replacing *mZP2* with *Izumo1* in pCAGGS with EcoRI |
| Izumo1 R XhoI | TCCATCTCGAGGGCCGCGTACG | Reverse primer for replacing *mZP2* with *Izumo1* in pCAGGS with XhoI |
| Izumo1 F NheI | TTATCGCTAGCGAATTCGGTCGACTGGATCC | Forward primer for cloning *Izumo1* into pCI::H2B-RFP and pCI::GFPnes vectors with NheI, and *Izumo1* mutants into pCAGGS with EcoRI |
| Izumo1 R SmaI | GTCCCCCGGGGCTCAATGGTGATGGTGATGATG | Reverse primer for cloning *Izumo1* into pCI::H2B-RFP and pCI::GFPnes vectors with SmaI |
| Juno F NheI | TAAGCTAGCCTCTTTGGCATCAGGAGGAGC | Forward primer for cloning *Juno* into pCI::H2B-RFP and pCI::GFPnes vectors with NheI |
| Juno R SmaI | ATACCCCGGGTGCCCCCAACATGAATAGCC | Reverse primer for cloning *Juno* into pCI::H2B-RFP and pCI::GFPnes vectors with SmaI |
| BlpI Flag F | TCAGCGACTACAAAGACGATGACGACAAGT | Forward oligo for tagging JUNO with a Flag tag using the BlpI site |
| BlpI Flag R | TGAACTTGTCGTCATCGTCTTTGTAGTCGC | Reverse oligo for tagging JUNO with a Flag tag using the BlpI site |
| GCS1 F NheI | CTAGCTAGCGGTACCATGGTGAACGCGATTTTAATG | Forward primer for cloning *GCS1/HAP2* into pCI::H2B-RFP and pCI::GFPnes vectors with NheI |
| GCS1 R SmaI | TCCCCCGGGCTAATGGTGATGGTGATGATGACC | Reverse primer for cloning *GCS1/HAP2* into pCI::H2B-RFP and pCI::GFPnes vectors with SmaI |
| GEX2 F KpnI | TCACAGGCCACCAAGCTTGGTACCATGGCGATTAAATTCGTTTCA  C | Forward primer for generating and cloning *Gex2-venus* into pGENE vector with KpnI |
| GEX2 R | GCTGCCCCCTCCACCTGACTGCTTATTCTGGTTGCCGGAAG | Reverse primer for generating *Gex2-venus* |
| Venus F | TCAGGTGGAGGGGGCAGCGGGGGGGGAGGTATGGTGAGCAAG GGCGAG | Forward primer for generating *Gex2-venus* |
| Venus R NotI | GTGACCTCGAGCGGCCGCTTACTTGTACAGCTCGTCCATGCC | Forward primer for generating and cloning *Gex2-venus* into pGENE vector with NotI |
| Izumo1-ecto-v5-R- SmaI | TCCCCCGGGCTACGTAGAATCGAGACCGAGGAGAGGGTTAGG GATAGGCTTACCTGGATTTTGAGCGACTGTAG | Reverse primer for cloning the ectodomain of Izumo1 with V5 tag and SmaI restriction enzyme site |
| Izumo1-W148A overlap F | ATGTCGCAGACTTTGATCGCTTGTCTTAAGTGCGAAAAG | Forward primer for cloning downstream of Izumo1^W148A^ by overlap pcr |
| Izumo1-W148A overlap R | CTTTTCGCACTTAAGACAAGCGATCAAAGTCTGCGACAT | Reverse primer for cloning upstream of Izumo1^W148A^ by overlap pcr |
| Izumo1-ΔIg overlap F | CGGAAATCCCTAGATTGTCCCCCAAAGCATTCAGAG | Forward primer for cloning downstream of Izumo1^ΔIg^ by overlap pcr |
| Izumo1-ΔIg overlap R | CTCTGAATGCTTTGGGGGACAATCTAGGGATTTCCG | Reverse primer for cloning downstream of Izumo1^ΔIg^ by overlap pcr |
| Izumo1-F28A-F | TGCATCAAATGTGACCAGGCTGTGACAGATGCGCTAAAG | Forward primer for cloning the second fragment of Izumo1^FWW^ mutant by overlap pcr |
| Izumo1-F28A-R | CTTTAGCGCATCTGTCACAGCCTGGTCACATTTGATGCA | Reverse primer for cloning the first fragment of Izumo1^FWW^ mutant by overlap pcr |
| Izumo1-WWAA-R | CTTTTGATGACGAAGCATAGCCAATAGTTCCTTTATAAAGAGCTC  TCCTTTTAAGTCACTGTCTGTAATACGCTTCAGATCCTTCAGAAA  ACTAGCGGTTGCTTGTTCCAGTGT | Reverse primer for cloning the second fragment of Izumo1^FWW^ mutant by overlap pcr |
| Izumo1-W113A-F | TTTATAAAGGAACTATTGGCTATGCTTCGTCATCAAAAG | Forward primer for cloning the third fragment of Izumo1^FWW^ mutant by overlap pcr |
| SmaI-His-R | TCCCCCGGGCTAATGGTGATGGTGATGATGACC | Reverse primer for cloning Izumo1 mutants |
| V5-SmaI R | TCCCCCGGGTCACGTAGAATCGAGACC | Reverse primer for cloning Izumo1 mutants into pCAGGS. |
| Izumo1-GFP EcoRI F | GCCGGGAATTCGTCGACTGG | Forward primer for cloning IZUMO1 GFP into pLVX-TetOne-Puro vector with EcoRI |
| Izumo1-GFP AgeI R | TCCCACCGGTAAACAGCTATGACCGCGGCC | Reverse primer for cloning IZUMO1 GFP into pLVX-TetOne-Puro vector with AgeI |
| IZUMO1-mCherry F | CCTTCCTGCGGCTTGTTCTCT | Forward primer for genotyping. For transgenic mice a 700 bp product is amplified. |
| IZUMO1-mCherry R | ATCAAGGTCTCAGAACTGTTCTCCCAAACC | Reverse primer for genotyping. For transgenic mice a 700 bp product is amplified. |
| CD9-GFP F | TGAACCGCATCGAGCTGAAGGG | Forward primer for genotyping. For transgenic mice a 700 bp product is amplified. |
| CD9-GFP R | GAATATCACCAAGAGGAACC | Reverse primer for genotyping. For transgenic mice a 700 bp product is amplified. |
