# Supplementary material for: A novel function for the sperm adhesion protein IZUMO1 in cell–cell fusion
Source: J Cell Biol. 2022 Nov 17;222(2):e202207147. doi: 10.1083/jcb.202207147 (PMC9671554; doi:10.1083/jcb.202207147)
Supplement: Table S3 — lists plasmids used for this study. [file JCB_202207147_TableS3.docx]

| **Table S3. Plasmids used for this study** | |
| --- | --- |
| **Plasmid** | **Origin** |
| pCI::H2B-RFP | (Williams et al., 2018), Addgene #92398 |
| pCI::GFPnes | (Moi et al., 2022) |
| myr-EGFP | (Dunsing et al., 2018) |
| pCMV6-AC-IZUMO1-GFP | Origene #MG222708 |
| Mouse ZP2 (JD#147) | (Liang et al., 1990), Addgene #14645 |
| pIZT::mZP2-V5-6HIS | This paper |
| pCAGGS::mZP2-V5-6HIS | This paper |
| pCAGGS::IZUMO1-V5-6HIS | This paper |
| pExpress1-JUNO | (Bianchi et al., 2014) |
| pExpress1-JUNO-flag | This paper |
| pcDNA3.1-EGFP-MBD-nls | (Yamagata et al., 2005) |
| pCI::AtGCS1/HAP2-V5::H2B-RFP | (Moi et al., 2022) |
| pCI::AtGCS1/HAP2-V5::GFPnes | This paper |
| pCI::JUNO::H2B-RFP | This paper |
| pCI::JUNO::GFPnes | This paper |
| pCI::IZUMO1-V5-6HIS::H2B-RFP | This paper |
| pCI::IZUMO1-V5-6HIS::GFPnes | This paper |
| pGene/V5-His | Thermo Fisher Scientific, Cat# K106001 |
| pSwitch | Thermo Fisher Scientific, Cat# K106001 |
| pRFPnes | (Avinoam et al., 2011) |
| Mouse E-cadherin GFP | (Truffi et al., 2014), Addgene #67937 |
| pGENE::mCherry-JUNO | (Nakajima et al., 2022) |
| pGENE::IZUMO1-Venus | (Nakajima et al., 2022) |
| pGENE::CeEFF-1 | (Valansi et al., 2017), Addgene #132961 |
| pGENE::AtGEX2-Venus | This paper |
| pCI::IZUMO1^ecto^-V5::H2B-RFP | This paper |
| pCI::IZUMO1^ecto^-V5::GFPnes | This paper |
| pCI::IZUMO1^ΔIg^-V5::H2B-RFP | This paper |
| pCI::IZUMO1^ΔIg^-V5::GFPnes | This paper |
| pCI::IZUMO1^W148A^-V5::H2B-RFP | This paper |
| pCI::IZUMO1^W148A^-V5::GFPnes | This paper |
| pCI::IZUMO1^FWW^-V5::H2B-RFP | This paper |
| pCI::IZUMO1^FWW^-V5::GFPnes | This paper |
| pMD2.G - VSV-G encoding plasmid | Didier Trono, Addgene #12259 |
| psPAX2 | Didier Trono, Addgene #12260 |
| pCAGGS::IZUMO1^ecto^-V5 | This paper |
| pCAGGS::IZUMO1^ΔIg^-V5 | This paper |
| pCAGGS::IZUMO1^W148A^-V5 | This paper |
| pCAGGS::IZUMO1^FWW^-V5 | This paper |
| pLVX-TetOne-Puro vector | Clontech Cat# 631849 |
| pLVX-TetOn-IZUMO1-GFP | This paper |

References

Avinoam, O., K. Fridman, C. Valansi, I. Abutbul, T. Zeev-Ben-Mordehai, U.E. Maurer, A. Sapir, D. Danino, K. Grünewald, J.M. White, et al.. 2011. Conserved eukaryotic fusogens can fuse viral envelopes to cells. Science. 332:589–592. 10.1126/science.1202333

Bianchi, E., B. Doe, D. Goulding, and G.J. Wright. 2014. Juno is the egg Izumo receptor and is essential for mammalian fertilization. Nature. 508:483–487. 10.1038/nature13203

Dunsing, V., M. Luckner, B. Zühlke, R.A. Petazzi, A. Herrmann, and S. Chiantia. 2018. Optimal fluorescent protein tags for quantifying protein oligomerization in living cells. Sci. Rep. 8:10634. 10.1038/s41598-018-28858-0

Liang, L.F., S.M. Chamow, and J. Dean. 1990. Oocyte-specific expression of mouse Zp-2: Developmental regulation of the zona pellucida genes. Mol. Cell. Biol. 10:1507–1515. 10.1128/mcb.10.4.1507-1515.1990

Moi, D., S. Nishio, X. Li, C. Valansi, M. Langleib, N.G. Brukman, K. Flyak, C. Dessimoz, D. de Sanctis, K. Tunyasuvunakool, et al. 2022. Discovery of archaeal fusexins homologous to eukaryotic HAP2/GCS1 gamete fusion proteins. Nat. Commun. 13:3880. 10.1038/s41467-022-31564-1

Nakajima, K.P., C. Valansi, D. Kurihara, N. Sasaki, B. Podbilewicz, and T. Higashiyama. 2022. Live imaging-based assay for visualising species-specific interactions in gamete adhesion molecules. Sci. Rep. 12:9609. 10.1038/s41598-022-13547-w

Truffi, M., V. Dubreuil, X. Liang, N. Vacaresse, F. Nigon, S.P. Han, A.S. Yap, G.A. Gomez, and J. Sap. 2014. RPTPα controls epithelial adherens junctions, linking E-cadherin engagement to c-Src-mediated phosphorylation of cortactin. J. Cell Sci. 127:2420–2432. 10.1242/jcs.134379

Valansi, C., D. Moi, E. Leikina, E. Matveev, M. Graña, L.V. Chernomordik, H. Romero, P.S. Aguilar, and B. Podbilewicz. 2017. Arabidopsis HAP2/GCS1 is a gamete fusion protein homologous to somatic and viral fusogens. J. Cell Biol. 216:571–581. 10.1083/jcb.201610093

Williams, R.M., U. Senanayake, M. Artibani, G. Taylor, D. Wells, A.A. Ahmed, and T. Sauka-Spengler. 2018. Genome and epigenome engineering CRISPR toolkit for in vivo modulation of cis-regulatory interactions and gene expression in the chicken embryo. Development. 145:dev160333. 10.1242/dev.160333

Yamagata, K., T. Yamazaki, M. Yamashita, Y. Hara, N. Ogonuki, and A. Ogura. 2005. Noninvasive visualization of molecular events in the mammalian zygote. Genesis. 43:71–79. 10.1002/gene.20158
