# Supplementary material for: A novel function for the sperm adhesion protein IZUMO1 in cell–cell fusion
Source: J Cell Biol. 2022 Nov 17;222(2):e202207147. doi: 10.1083/jcb.202207147 (PMC9671554; doi:10.1083/jcb.202207147)

Source Data

Uncropped Western blot images from Figure 4.
Western blot probed with anti-V5 or anti-actin antibodies. The sections indicated by red dashed squares are used in Figure 4C.

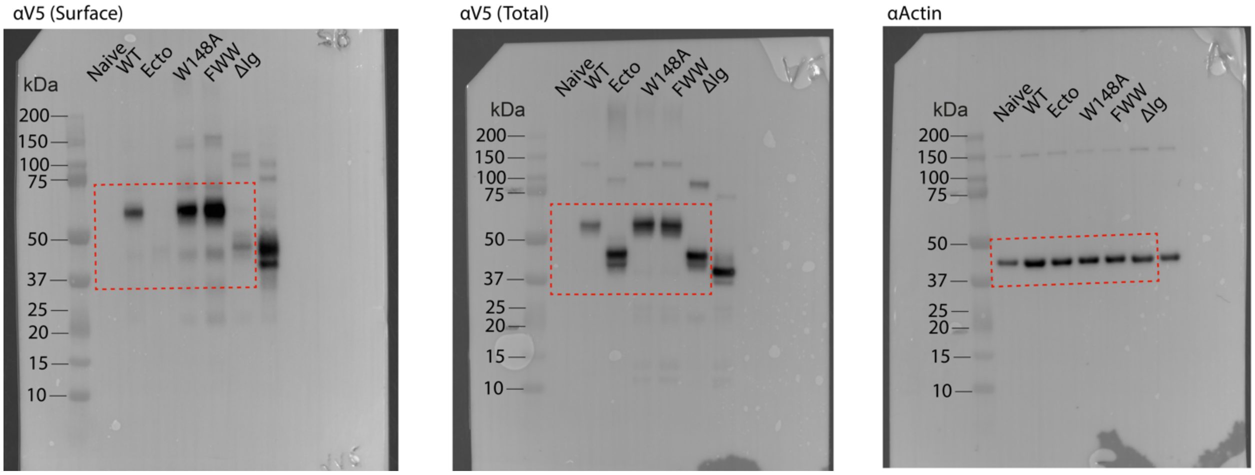

Supplement: SourceData F4 — contains original blots for Fig. 4. [file JCB_202207147_SourceDataF4.pdf]
